# Transmission of *Mycobacterium tuberculosis* in a Rural Community, Arkansas, 1945–2000

**DOI:** 10.3201/eid0811.020299

**Published:** 2002-11

**Authors:** Jennifer A. Dillaha, Zhenhua Yang, Kashef Ijaz, Kathleen D. Eisenach, M. Donald Cave, Frank J. Wilson, William W. Stead, Joseph H. Bates

**Affiliations:** * University of Arkansas for Medical Sciences, Little Rock, Arkansas, USA; † Medical Research Service, Central Arkansas Veterans Healthcare System, Little Rock, Arkansas, USA; ‡ Arkansas Department of Health, Little Rock, Arkansas, USA

**Keywords:** tuberculosis, *Mycobacterium tuberculosis*, endemic, molecular epidemiology, DNA fingerprinting, genotyping

## Abstract

A cluster of tuberculosis cases in a rural community in Arkansas persisted from 1991 to 1999. The cluster had 13 members, 11 linked epidemiologically. Old records identified 24 additional patients for 40 linked case-patients during a 54-year period. Residents of this neighborhood represent a population at high risk who should be considered for tuberculin testing and treatment for latent tuberculosis infection.

Research based on IS*6110*-based DNA fingerprinting provides insights into the transmission patterns of *Mycobacterium tuberculosis*. Population-based studies from San Francisco (1) and New York City (2) showed a high incidence of DNA fingerprint clustering that reflected recent transmission. Further studies in urban populations found that clusters occurred within geographic areas, demographic groups, or social networks (3); a high proportion of recently transmitted disease was propagated among a population of difficult-to-serve patients (3). Traditional contact investigations did not reliably identify patients who were infected with the same *M. tuberculosis* strain (4,5).

In contrast to studies in urban areas, few investigations of transmission patterns in rural populations have been undertaken. A study conducted in Arkansas suggests that DNA fingerprint clustering in stable rural populations may result from remote rather than recent transmission (6). However, studies have been limited by short time periods (2–3 years), which made it difficult to assess the contribution of remote transmission to clustering. In addition, some unique strains may result from recent rather than remote transmission, and they would form clusters over a sufficient time span.

## The Study

In this study, DNA fingerprints that were based on IS*6110* genotyping were used. This method of strain typing provides stable restriction fragment length polymorphism patterns that are useful in epidemiologic investigations to determine transmission between persons. A cluster is defined as two or more isolates with fingerprints that matched 100% by software analysis using a BioImage Whole Band Analyzer (Millipore, Ann Arbor, MI). Clustering refers to a set of isolates that have the same fingerprint pattern or genotype.

During our investigation of nursing home–related tuberculosis (TB) in Arkansas, we used a database of all DNA fingerprints obtained from Arkansas isolates in 1991–1999. A large, persistent cluster in a rural county (county Q, population 21,000) was identified. This cluster was an extension of one described previously (6). The strain variation in county Q was studied to determine whether this cluster could represent an endemic strain. For this purpose, DNA fingerprints obtained from Arkansas isolates during 1993–1999 were examined. (DNA fingerprints were available for 85% of all culture-confirmed cases in county Q during that period.) Public health and medical records identified epidemiologic links among members of the cluster and other TB case-patients in the county.

Sixteen cases of TB were reported from county Q during 1993–1999. Of the 13 culture-positive patients, we obtained DNA fingerprints for 11. Four distinct patterns were observed. The most common pattern was in isolates from eight patients in the county Q cluster. Isolates from three patients from that county did not match any other county’s isolates, but each patient’s isolate matched at least one from another area in Arkansas. DNA fingerprints were not obtained for 2 of the 13 culture-proven cases. One was epidemiologically linked to a case that had one of the unique fingerprints. The second (patient 35, Figure) was epidemiologically linked to the county Q cluster.

**Figure:**
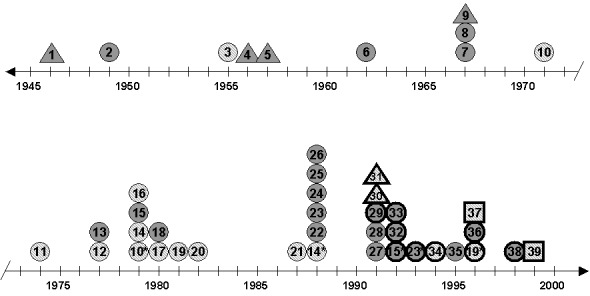
Timeline of tuberculosis cases in a large, epidemiologically linked group of persons living in rural Arkansas. Circle, epidemiologically linked case-patient**,** resided in neighborhood X; triangle, epidemiologically linked patient who did not reside in neighborhood X; square, nonepidemiologically linked case-patient; gray, resided in village Z; shaded, did not reside in village Z; bold circle, bold triangle, or bold square, DNA fingerprint available; asterisk, reactivation of prior disease.

Three persons also had negative cultures during this period. They included two siblings identified as contacts to their father, who had recently immigrated from Africa, and a resident of another town in county Q who had no epidemiologic links.

The county Q cluster had a 15-band fingerprint and spanned 9 years (1991–1999). The cluster contained 13 members; 11 had epidemiologic links. Nine of the 11 linked members resided in the same area (neighborhood X) of a rural community (town Y, population 3,500). They frequented the same neighborhood bars associated with extensive transmission in the past. Three of the nine persons (patients 15, 19, and 23; Figure) had culture-confirmed TB that was associated with bar-related outbreaks in 1979 and 1988. Compliance with appropriately prescribed therapy was poor, according to health-care providers. One of the three persons (patient 19, Figure) required incarceration to complete his treatment. All three persons were HIV negative and probably had reactivation of prior disease. Their initial isolates were not available for comparison.

Eight of the nine cluster members from neighborhood X were men; the woman (patient 32, Figure) was the girlfriend of a bar owner. She had diabetes mellitus, renal failure, HIV infection, and smear-negative pulmonary TB. Five of the eight men had smear-positive sputum; all were HIV negative. Two of the men (patients 19 and 34, Figure) had been inmates in the county jail; one (patient 34, Figure) also had been in a state prison.

Six of the cluster members from neighborhood X were originally from a farming community (village Z, population 200) 15 miles from town Y. Two of these six patients (patients 23 and 36, Figure) are brothers, and family members of five had TB in the past.

Two of the linked cluster members did not reside in town Y. They were medical personnel infected during an autopsy on a linked patient (patient 29, Figure), who died of unrecognized, disseminated TB (7). This patient had returned to Arkansas recently after living in California for many years. His medical records indicated that infection likely occurred after his return to town Y. The two medical personnel were not usual cases; induced sputum grew *M. tuberculosis* and verified that transmission occurred during the autopsy. Symptoms did not develop in either person.

Two cluster members had no epidemiologic links. The first, a 78-year-old nursing home resident (patient 37, Figure), resided in neighborhood X before entering the nursing home in town Y in March 1995. He had a negative two-step tuberculin skin test on admission. TB was diagnosed in August 1996 by a culture from a shoulder aspirate. His chest x-ray showed an abnormality, and his tuberculin skin-test result was positive at the time of diagnosis. During the source case investigation, we learned that the tuberculin skin tests of four other residents converted after they were admitted. No source case was identified.

The second unlinked cluster member was a 55-year-old woman (patient 39, Figure) who was employed at the county Q jail; she resided in a different community. She was a nurse and had received annual tuberculin skin tests through October 1997 that were negative. After she had worked at the jail for a year, cavitary TB was diagnosed in December 1998. Her tuberculin skin test was positive at the time of diagnosis. No source case was identified.

 A review of health department records for county Q identified 24 other persons who had epidemiologic links to the county Q cluster. TB was diagnosed for all of these persons before DNA fingerprinting was available (with the exception of patient 35, Figure). Thus, 40 related cases occurred among 35 patients during 54 years in this community. The earliest documented diagnosis (patient 1, Figure) was in 1946.

All but one of the members of this large group are African-Americans; they resided either in neighborhood X or in village Z. A white teenage boy (patient 3, Figure), who resided in neighborhood X, was the exception. The ages of patients at the time of diagnosis ranged from 2 to 80 years (average 47 years). Seven patients were >65 years old. Numerous children were exposed during this 54-year period; they were treated for TB but were not included in the group. TB caused at least three deaths in this large, epidemiologically related group, although all isolates remained drug-sensitive.

## Conclusions

This endemic strain of TB appears to have persisted in this rural community for at least 54 years despite determined TB-control efforts. Repetitive cycles of transmission and infection occurred and were followed by reactivation or progression to disease. The actual transmission path could not be delineated unequivocally. Whether an episode was recrudescent or primary disease was often indeterminable.

The findings of this rural study are similar to those of urban studies. The county Q epidemiologically related group represents a difficult-to-serve population. Many of the members were poor adherents to therapy. They were distrustful of the public health system and resistant to care offered through the health department. In addition, alcohol and drug abuse, other illicit activities, and mental illness played major roles in the transmission and adherence dynamics of this group.

Contact tracing with recommendation for treatment of latent TB infection among persons with positive tuberculin skin-test results has been unsuccessful in eliminating TB in this population. Reasons for this failure may include concerns about anonymity and reluctance of persons to disclose contacts who are part of social networks involved in illicit activity. Alternative measures to reduce TB transmission are needed. Case-finding and screening for latent infection on the basis of geographic location rather than personal contacts are important options (8). Such a strategy would incorporate the relevant social networks without stigmatization and identify persons who would be missed otherwise. An effective partnership between the health department and this high-risk neighborhood is crucial to eliminating TB from this community (9).

In contrast to urban areas of the United States, where case rates are 2.5 times higher than the national rate, the rural population in Arkansas has a higher case rate than the urban population. (The 1999 statewide case rate was 7.1 per 100,000. The aggregate rate for counties in metropolitan statistical areas was 5.6, and the aggregate rate for rural counties was 8.5.) In this setting, identification of TB-endemic strains followed by case-finding and screening for latent infection in defined geographic areas may play an important role in the successful elimination of this disease in the United States.
